# Sex-specific genetic determinants for arterial stiffness in Dahl salt-sensitive hypertensive rats

**DOI:** 10.1186/s12863-015-0324-7

**Published:** 2016-01-11

**Authors:** Julius L. Decano, Khristine A. Pasion, Nicole Black, Nicholas J. Giordano, Victoria L. Herrera, Nelson Ruiz-Opazo

**Affiliations:** Department of Medicine, Whitaker Cardiovascular Institute, Boston University School of Medicine, 700 Albany Street, W-609, Boston, MA 02118 USA

**Keywords:** Genetic linkage, QTL, Arterial stiffness, Pulse wave velocity, Arterial strain, Dahl rat

## Abstract

**Background:**

Arterial stiffness is an independent predictor of cardiovascular outcomes in hypertensive patients including myocardial infarction, fatal stroke, cerebral micro-bleeds which predicts cerebral hemorrhage in hypertensive patients, as well as progression to hypertension in non-hypertensive subjects. The association between arterial stiffness and various cardiovascular outcomes (coronary heart disease, stroke) remains after adjusting for age, sex, blood pressure, body mass index and other known predictors of cardiovascular disease, suggesting that arterial stiffness, measured via carotid-femoral pulse wave velocity, has a better predictive value than each of these factors. Recent evidence shows that arterial stiffening precedes the onset of high blood pressure; however their molecular genetic relationship (s) and sex-specific determinants remain uncertain. We investigated whether distinct or shared genetic determinants might underlie susceptibility to arterial stiffening in male and female Dahl salt-sensitive rats. Thus, we performed a genome-wide scan for quantitative trait loci (QTLs) affecting arterial stiffness in six-week old F2 (Dahl S x R)-intercross male and female rats characterized for abdominal aortic pulse wave velocity and aortic strain by high-resolution ultrasonography.

**Results:**

We detected five highly significant QTLs affecting aortic stiffness: two interacting QTLs (*AS-m1* on chromosome 4 and *AS-m2* on chromosome16, LOD 8.8) in males and two distinct interacting QTLs (*AS-f1* on chromosome 9 and *AS-f2* on chromosome11, LOD 8.9) in females affecting pulse wave velocity. One QTL (*AS-1* on chromosome 3, LOD 4.3) was found to influence aortic strain in a sex-independent manner. None of these arterial stiffness QTLs co-localized with previously reported blood pressure QTLs detected in equivalent genetic intercrosses.

**Conclusions:**

These data reveal sex-specific genetic determinants for aortic pulse wave velocity and suggest distinct polygenic susceptibility for arterial stiffness and salt-sensitive hypertension in Dahl rats based upon reported blood pressure QTLs in equivalent (Dahl S x R)-intercrosses.

## Background

Several studies support a genetic component contributing to arterial stiffness. Heritability for pulse wave velocity (PWV) in young adults in the Georgia Cardiovascular Twin Study was about 0.43 and 0.53 for aorto-radial and aorto-dorsalis-pedis PWV respectively [1]. In the Framingham Heart Study cohort a heritability of 0.4 was reported [2]. Genetic linkage studies in the Framingham cohort reported several regions of linkage, albeit suggestive rather than significant, for carotid femoral PWV on chromosomes 2, 7, 13 and 15 (LOD scores > 2.0) [2]. Analysis of common polymorphisms in several candidate genes have been performed, however only *CYP11B2* and *NOS3* have been confirmed by genome-wide linkage studies [3] as being associated with arterial stiffness. While these observations demonstrate the heritability and the quantitative trait nature of arterial stiffness, they also illustrate the complexity of arterial stiffness, and hence the ensuing difficulties in the study of its genetic basis in the general population. These suggest the need for systematic animal model studies in dissecting causal paradigms for susceptibility.

To date, however, no genome wide studies of arterial stiffness in the context of polygenic hypertension have been done in animal models. Gaugier and collaborators identified a QTL controlling elastin content in rat aorta [4]; however, their study was done in non-hypertensive rat strains: Brown Norway rat strain and the LOU rat strain. More specifically, the Brown Norway rat strain shows an aortic elastin deficit by 5.8 % by dry-weight compared with the inbred LOU rat, and is also highly susceptible to spontaneous rupture of the internal elastic lamina in its abdominal aorta – however Brown Norway rats are not hypertensive. Additionally, analysis of the aortic elastic network and related phenotypes in seven inbred rat strains was reported; however, these were all done in non-hypertensive rat strains [5].

Given that arterial stiffness precedes hypertension in a Dahl salt-sensitive hypertensive stroke-prone rat model [6] as it does in humans; this experimental model system allows the systematic dissection as to whether common or distinct genetic determinants underlie hypertension and arterial stiffness. Having performed QTL analyses for blood pressure in male and female F2 ([Dahl S] Dahl salt-sensitive x [Dahl R] Dahl salt-resistant)-intercrosses [7, 8] we performed a total genome scan for QTLs affecting aortic Pulse Wave Velocity (PWV) and aortic Strain as quantitative measures of arterial stiffness using an F2 (Dahl S x R)-intercross male and female rat populations phenotyped for aortic PWV and aortic Strain at six weeks of age prior to the onset of hypertension. Results were then compared to blood pressure QTLs detected in previous genome scans performed in male [7] and female F2 [Dahl S x R]-intercross rats [7, 8]. Data demonstrate that distinct QTLs underlie male and female arterial stiffness in Dahl rats. Moreover, none of the arterial stiffness QTLs co-localized with blood pressure QTLs detected in genetically equivalent F2 [Dahl S x R]-intercrosses indicating distinct genetic mechanisms underlying arterial stiffness and hypertension in Dahl rats.

## Results and discussion

### Study cohorts

To investigate the genetic determinants that contribute to arterial stiffness we performed a genome scan for QTLs affecting aortic (between superior mesenteric artery and left renal artery) PWV and strain in an F2 (Dahl S x R)-intercross male and female rat populations phenotyped for aortic PWV and aortic strain by high-resolution ultrasonography. We implemented the following experimental design: F2 hybrids were produced from dams on Purina 5001 (0.4 % NaCl) during gestation and lactating and maintained on Purina 5001 at weaning. Ultrasound measurements of abdominal aortic PWV and strain were done at 6 weeks of age following identical procedures. Rather than carotid-femoral PWV measurements, we measured aortic PWV between two points: below the superior mesenteric artery and above the left renal artery. This allows reproducible measurements of aortic PWV, between two points of the same artery guided by clear anatomic landmarks. We measured aortic PWV and aortic Strain in 142 male and 140 female subjects. The cohort was genotyped with 110 informative markers for our (Dahl S x R) intercross with an average density of 21.7 Mbp distance between two markers.

**Fig. 1:**
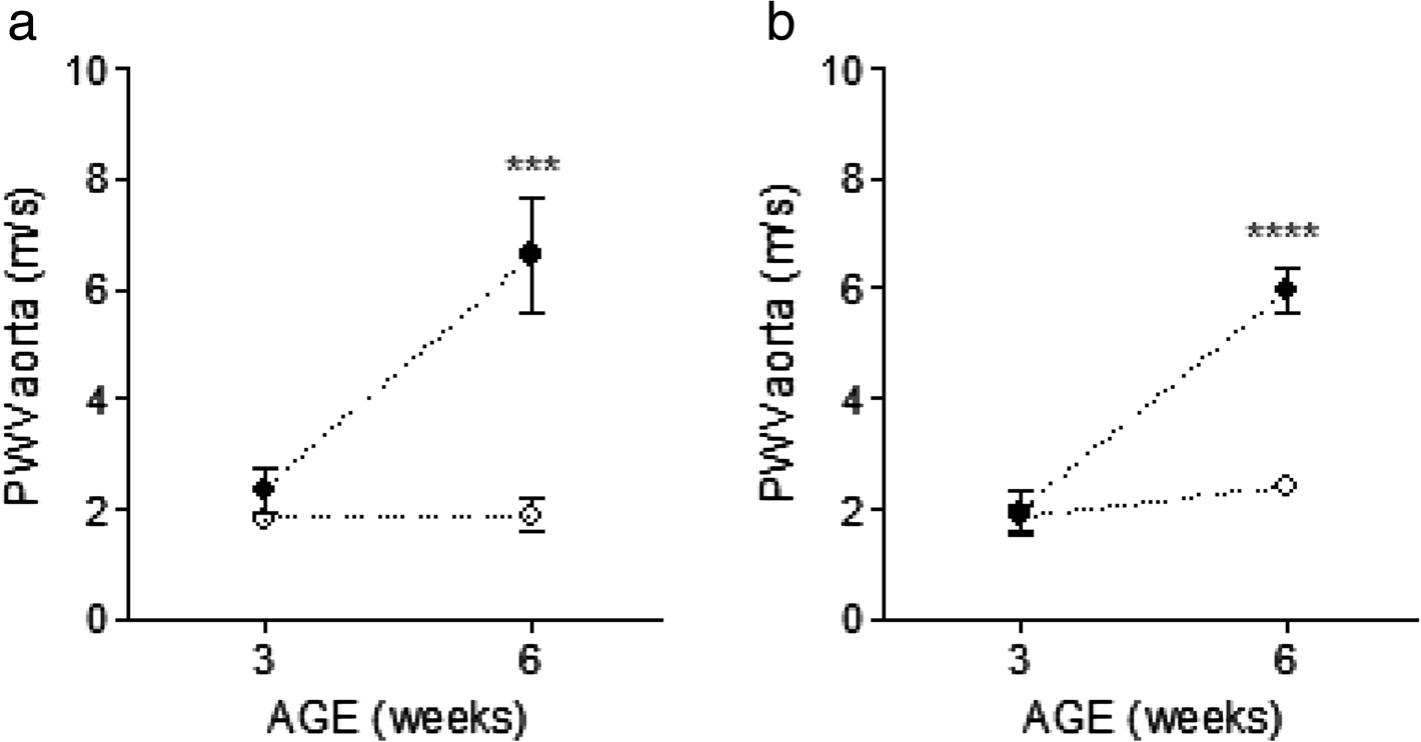
Development of arterial stiffness in Dahl S and Dahl R rats measured by pulse wave velocity (PWV). Aortic PWV (PWV_aorta_) was measured in male (**a**) and female (**b**) Dahl S (black circle) and Dahl R (open circle) rats at 3 and 6 weeks of age. Dahl S males at 3 weeks of age, *n* = 4; Dahl S males at 6 weeks of age, *n* = 4; Dahl S females at 3 weeks of age, *n* = 5; Dahl S females at 6 weeks of age, *n* = 6; Dahl R males at 3 weeks of age, *n* = 4, Dahl R males at 6 weeks of age, *n* = 6; Dahl R females at 3 weeks of age, *n* = 6, Dahl R females at 6 weeks of age, *n* = 6. Values are means ± s.e.m. ^***^ *P* < 0.001, ^****^ *P* < 0.00001 (One Way ANOVA followed by Holm-Sidak Test for multiple comparisons)

In order to determine the earliest developmental age for arterial stiffness measurements and potentially eliminate blood pressure as a confounder in our ultrasound measurements we first analyzed the time course of development of arterial stiffness in parental Dahl S and Dahl R rats. As shown in Fig. 1 aortic PWV was significantly elevated in Dahl S male (Fig. 1a, *P* < 0.001) and Dahl S female (Fig. 1b, *P* < 0.00001) subjects when compared with corresponding Dahl R subjects at 6 weeks of age. The fact that the phenotypic differences in arterial stiffness measures are detected at 6 weeks of age in animals maintained on a 0.4 % NaCl regular rat chow diet, prior to the onset of high blood pressure, eliminates hypertension as a potential confounder in our genetic linkage study. The mean aortic PWV values in both male and female F2 populations fall between the parental mean values (Table 1) suggesting a co-dominant effect for both Dahl S and Dahl R alleles on the trait. In contrast, the mean aortic strain values for the F2 male and female cohorts approached the value obtained for the Dahl R rats (Table 1) indicating a dominance effect of the alleles for the trait (recessive for Dahl S and dominant for Dahl R respectively). This differential trend also indicates that aortic PWV is a distinct trait from aortic strain.

**Table 1:** Aortic Pulse Wave Velocity and aortic strain in Dahl S, Dahl R and F2 intercross populations at six weeks of age

Cohort	n	PWV_aorta_	STR_aorta_
*Males*			
Dahl R	6	2.56 ± 0.42	0.179 ± 0.012
Dahl S	4	7.96 ± 1.88	0.142 ± 0.008
F2 intercross	142	4.72 ± 2.09	0.176 ± 0.002
*Females*			
Dahl R	6	2.51 ± 0.92	0.185 ± 0.009
Dahl S	6	6.42 ± 0.54	0.104 ± 0.022
F2 intercross	140	4.77 ± 1.95	0.177 ± 0.002
*Statistical testing*			
Dahl S vs Dahl R, M		*P* < 0.001	*P* = 0.030
Dahl S vs F2, M		*P* = 0.004	*P* = 0.008
Dahl R vs F2, M		*P* = 0.013	*P* = n.s.
Dahl S vs Dahl R, F		*P* = 0.001	*P* < 0.001
Dahl S vs F2, F		*P* = 0.040	*P* < 0.001
Dahl R vs F2, F		*P* = 0.009	*P* = n.s.

Data is presented for populations and traits informative for QTL detection (male, female F2 populations phenotyped for PWV and STR). Values are Mean ± standard deviation; *PWV* pulse wave velocity, *STR* aortic proximal strain, *M* male, *F* female; Animals were maintained on Purina (0.4 % NaCl) rat diet from inception; *P*, Two Way ANOVA followed by Holm-Sidak test for multiple comparisons

### Total genome scan analysis

**Fig. 2:**
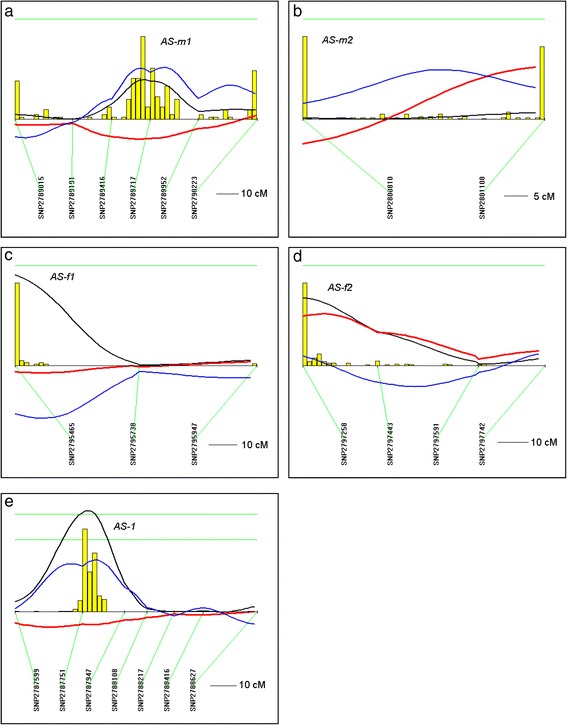
QTLs for arterial stiffness (*AS*) in F_2_ (Dahl S x R)-intercross rats. Chromosomes with interacting QTLs in male (**a**, **b**) and female (**c**, **d**) F2 cohorts and a chromosome with a highly significant QTL detected in the combined female + male F2 cohorts were analyzed by interval mapping with bootstrap resampling method to estimate a confidence interval (QTXb19 Map Manager): Panel **a**, chromosome 4 (*AS-m1*); **b**, chromosome 16 (*AS-m2*); **c**, chromosome 9 (*AS-f1*); **d**, chromosome 11 (*AS-f2*) and **e**, chromosome 3 (*AS-1*). Yellow histograms represent the bootstrap-based confidence intervals for the detected QTLs. For a histogram with single peak, widths define the confidence interval for the QTL. Orientation of chromosomes: left → right starting from lowest Mbp. Horizontal green line for **a**-**d** mark LOD value for significant linkage (LOD ≥ 3.0). Horizontal green lines for panel **e** from top to bottom: highly significant LOD ≥ 4.3; significant LOD ≥ 3.0. Regression coefficient for additive effect (*red* lines); regression coefficient for dominance effect (*blue* lines); LOD (*black* lines)

We next performed a total genome scan for QTLs affecting aortic PWV using 6 weeks-old F2 (Dahl S x R)-intercross male and female rats. Marker regression analysis did not detect any significant PWV QTL in both male and female cohorts, only suggestive QTLs: two in males and three in females (Table 2). However, subsequent analysis for interactive effects on aortic PWV reveals several interacting-loci affecting PWV that fulfilled the criteria for significant gene interaction (Fig. 2 and Table 3): *AS-m1* (on chromosome 4) and *AS-m2* (on chromosome 16) in the male cohort (LOD 8.8, Table 3) and *AS-f1* (on chromosome 9) and *AS-f2* (on chromosome 11) in the female population (LOD 8.9, Table 3). Similarly, sex-specific genetic linkage analysis for strain did not identify significant QTLs. However, QTL analysis on the combined male and female populations detected a highly significant QTL (*AS-1* on chromosome 3, LOD 4.3, Table 3) affecting aortic strain indicating that it influences aortic strain in a sex-independent manner.

**Table 2:** QTLs with suggestive linkage for arterial stiffness in F2 (Dahl S x R)-intercross rats

Chr	Reference marker	Location (Mbp)	LOD	Trait
*Males*				
5	SNP2790733	55.43	2.3	PWV_aorta_
20	SNP2803750	48.28	2.0	PWV_aorta_
*Females*				
9	SNP2795465	30.75	2.8	PWV_aorta_
11	SNP2797258	23.12	2.1	PWV_aorta_
15	SNP2800195	50.59	2.1	PWV_aorta_

*QTL* quantitative trait locus, *Chr* chromosome, *PWV*_*aorta*_ Pulse Wave Velocity in aorta, *LOD* logarithm of the odds score derived from the likelihood ratio statistic using a factor of 4.6. For marker regression analysis significance was determined from 2000 permutations on data set: Highly significant LOD ≥ 4.3, Significant LOD ≥ 3.0and Suggestive linkage LOD ≥ 2.0. SNP locations on rat genome as per NCBI Rat Genome Assembly Annotation Release 105

**Table 3:** Arterial stiffness (AS) QTLs detected by marker regression and interaction analysis in F2 (Dahl S x R)-intercross rats

Chr	QTL	Locus A (Mbp)	Main A	Chr	QTL	Locus B (Mbp)	Main B	LOD T	LOD I	Trait (F2)
4	*AS-m1*	SNP2789717 (173.96)	1.2	16	*AS-m2*	SNP2800810 (15.73)	0.1	8.8	7.6	PWV_aorta_ (M)
9	*AS-f1*	SNP2795465 (30.75)	2.8	11	*AS-f2*	SNP2797258 (23.12)	2.1	8.9	4.0	PWV_aorta_ (F)
3	*AS-1*	SNP2787751 (50.64)	4.3							STR_aorta_ (M + F)

*QTL* quantitative trait locus, *Chr* chromosome, *PWV*_*aorta*_ Pulse Wave Velocity in aorta, *STR*_*aorta*_ strain in aorta, *M* F2 male cohort, *F* F2 female cohort, *LOD* logarithm of the odds score derived from the likelihood ratio statistic using a factor of 4.6. For marker regression analysis significance was determined from 2000 permutations on data set: LOD 4.3 highly significant. Only interactive loci that exhibited a LOD > 8.0 (*P* < 10^−5^) for the total effect and a LOD > 4.0 (*P* < 0.01) for the interactive effect are presented. Main A and Main B refer to the specific LOD for A and B main (single locus) effects respectively. LOD T, LOD for total effect; LOD I, LOD for the interaction. SNP locations on rat genome as per NCBI Rat Genome Assembly Annotation Release 105

### Comparison of arterial stiffness and blood pressure QTLs

We identified five QTLs influencing arterial stiffness in our F2 (Dahl S x R) intercross genetic analysis, four affecting aortic PWV (on chromosomes 4, 16, 9 and 11) and one influencing aortic strain (on chromosome 3). Remarkably, none of the arterial stiffness QTLs co-localizes with previously reported blood pressure (BP) QTLs found in the same F2 (Dahl S x R) genetic intercrosses (Table 4). These data suggest the existence of distinct genetic determinants for salt-sensitive hypertension and arterial stiffness, which is consistent with the temporal dissociation of onset of arterial stiffness and hypertension observed in Dahl salt-sensitive rats [6].

**Table 4:** QTLs for blood pressure and arterial stiffness in F2 (Dahl S x R) intercross rats

QTL	Trait	F2 cohort	Chr: location	Reference
*AS-1*	STR_aorta_	M + F	Chr3: 40–60 Mbp	
*AS-m1*	PWV_aorta_	M	Chr4: 163–183 Mbp	
*AS-m2*	PWV_aorta_	M	Chr16: 6–26 Mbp	
*BP-m1*	SBP	M	Chr1: 142–162 Mbp	[7]
*BP-m2*	SBP	M	Chr1: 189–209 Mbp	[7]
*BP-m3*	SBP	M	Chr20: 42–62 Mbp	[7]
*BP-m4*	SBP	M	Chr2: 194–214 Mbp	[7]
*BP-m5*	SBP	M	Chr11: 34–54 Mbp	[7]
*AS-f1*	PWV_aorta_	F	Chr9: 15–35 Mbp	
*AS-f2*	PWV_aorta_	F	Chr11: 13–33 Mbp	
*BP-f1*	SBP	F	Chr5: 135–155 Mbp	[7]
*BP-f2*	SBP	F	Chr5: 103–123 Mbp	[7]
*BP-f3*	SBP	F	Chr12: 4–24 Mbp	[7]
*BP-f4*	SBP	F	Chr2: 179–199 Mbp	[7]
*BP-f5*	SBP	F	Chr2: 96–116 Mbp	[7]
*BP-f6*	SBP	F	Chr1: 146–166 Mbp	[7]
*ixBP-f1*	SBP	F	Chr1: 189–209 Mbp	[7]
*ixBP-f2*	SBP	F	Chr3: 115–135 Mbp	[7]
*ixBP-f3*	SBP	F	Chr14: 63–83 Mbp	[7]
*BP-pm1*	SBP	pmF	Chr13: 73–93 Mbp	[8]
*BP-pm2*	SBP	pmF	Chr11: 53–73 Mbp	[8]
*BP-pm3*	DBP	pmF	Chr2: 254–270 Mbp	[8]
*BP-pm4*	DBP	pmF	Chr4: 164–184 Mbp	[8]
*BP-pm5*	SBP	pmF	Chr15: 18–38 Mbp	[8]

*QTL* quantitative trait locus, *Chr* chromosome, *PWV*_*aorta*_ Pulse Wave Velocity in aorta, *STR*_*aorta*_ strain in aorta, *M* F2 male cohort, *F* F2 female cohort, *pmF* F2 post-menopausal female cohort, *BP* blood pressure, *AS* arterial stiffness, *Mbp* mega base pairs

Although further QTL-gene mapping needs to be done, inspection of the rat chromosomal regions spanning the arterial stiffness QTLs reveals potential candidate genes based on vascular biology paradigms (Table 5). For QTL *AS-1* on chromosome (Chr) 3 [40–60 Mbp], the Activin A receptor, Type 1 (ACVR1) at position 44.6 Mbp could underlie the *AS-1* effect on aortic strain in our (Dahl S x R) intercross. ACVR1 is a receptor for TGF-β1, a multifunctional cytokine that influences the vascular wall via actions on endothelial cells and modulation of the extracellular matrix [9–14]. TGF-β1 is a pro-fibrosis cytokine [11] and has been associated with fibrosclerotic development in varicose vessel walls [15]. Within the *AS-f1* region on Chr. 9 (15–35 Mbp), the Vascular endothelial growth factor A (VEGF-A) gene resides at position 17.4 Mbp. VEGF-A is a valid candidate gene contributing to increased PWV since VEGF-A is increased in pulmonary microvascular endothelial cells subjected to increased flow pulsatility, a parameter associated with increased arterial stiffness in vivo [16]. Scanning of the *AS-f2* QTL in the Chr. 11 (13–33 Mbp) region detects ADAMTS1 (A Disintegrin And Metalloproteinase [ADAM] with thrombospondin type 1 motif, 1) at coordinate 25.4 Mbp as candidate. ADAMTS1 has been implicated in arterial remodeling in multiple systems [17–19] and has been shown to be responsive to wall shear stress [18, 19].

**Table 5:** Candidate genes for arterial stiffness QTLs detected with significant linkage in F2 (Dahl S x R) rat intercross

QTL	QTL-location	Trait	Symbol	Location	Description
*AS-1*	Chr3: 40–60 Mbp	Strain	ACVR1	44.6 Mbp	Activin A receptor, Type 1
*AS-m1*	Chr4: 163–183 Mbp	PWV			
*AS-m2*	Chr16: 6–26 Mbp	PWV			
*AS-f1*	Chr9: 15–35 Mbp	PWV	VEGF A	17.4 Mbp	Vascular endothelial growth factor A
*AS-f2*	Chr11: 13–33 Mbp	PWV	ADAMTS 1	25.4 Mbp	A Disintegrin And Metalloproteinase (ADAM) with thrombospondin type 1 motif, 1

*QTL* quantitative trait locus, *[AS-m1]-[AS-m2]* are male-specific interacting QTLs, *[AS-f1]-[AS-f2]* are female-specific interacting QTLs, *Chr* chromosome, *Mbp* mega base pairs; Strain, aortic strain, *PWV* aortic pulse wave velocity. QTL and gene locations on rat genome as per NCBI Rat Genome Assembly Annotation Release 105

*AS-f1* and *AS-f2* are female-specific interacting QTLs affecting aortic pulse wave velocity. The finding that ADAMTS1 inhibits endothelial cell proliferation by direct binding and sequestration of VEGF-A [20] is consistent with our hypothesis that ADAMTS1 and VEGF-A might underlie the interacting effect of *AS-f1* and *AS-f2* on aortic PWV. Although additional fine mapping studies are necessary to substantiate candidate genes within detected QTL regions influencing arterial stiffness, these candidate gene localizations in QTLs with significant linkage integrate genetic factors with concordant pathophysiological paradigms of arterial stiffness.

Previous human genetic association studies have reported several candidate genes for loci associated with arterial stiffness (Table 6). However, only one out of ten loci was detected at a significant level with nine of them being suggestive for the association implying a degree of uncertainty in the association for these loci. Moreover, none of these candidate genes localize to syntenic regions harboring the detected rat arterial stiffness QTLs.

**Table 6:** Candidate genes for loci associated with arterial stiffness (cfPWV) in humans

Cohort	Sig	H-Position	Symbol	Description	Rat Position
Framingham	Suggestive	Chr15: 90.9 Mbp	FURIN	Paired basic amino acid cleaving enzyme	Chr1: 142.2 Mbp
Framingham	Suggestive	Chr15: 98.6 Mbp	IGF1R	Insulin-like growth factor-1 receptor	Chr1: 128.9 Mbp
Framingham	Suggestive	Chr15: 99.6 Mbp	MEF2A	Myocyte-specific enhancer factor 2A	Chr1: 128.4 Mbp
Framingham	Suggestive	Chr15: 101.2 Mbp	CHSY1	Chondroitin synthase 1	Chr1: 127.0 Mbp
Framingham	Suggestive	Chr15: 101.3 Mbp	PACE4	Proprotein convertase subtilisin/kexin type 6	Chr1: 126.7 Mbp
Framingham	Suggestive	Chr2: 70.7 Mbp	ADD2	β-adducin	Chr4: 117.7 Mbp
Framingham	Suggestive	Chr2: 75.0 Mbp	TACR1	Neurokinin-1 receptor	Chr4: 113.2 Mbp
Framingham	Suggestive	Chr2: 96.1 Mbp	ADRA2B	α-2B adrenergic receptor	Chr3: 119.8 Mbp
Framingham	Suggestive	Chr7: 22.7 Mbp	IL6	Interleukin-6	Chr4: 3.0 Mbp
Europe	Significant	Chr14: 99.2 Mbp	3′-BCL11B gene desert	3′-B-cell CLL/lymphoma 11B (zinc finger protein) gene desert	Chr6: 131.9 Mbp

*cfPWV* carotid-femoral pulse wave velocity, *Sig* significance, *Chr* chromosome, *Mbp* mega base pairs, *H-Position* human position as per NCBI Human Genome Assembly Annotation Release 106; Rat position as per NCBI Rat Genome Assembly Annotation Release 105. Framingham cohort (1480 participants) as reported by [2]; European cohort (20634 participants) as reported by [32]

## Conclusions

Altogether, our study demonstrates the involvement of distinct genetic determinants for aortic PWV and aortic strain in Dahl rats. In addition, based upon reported BP QTLs in equivalent intercrosses [7, 8] and our present findings, the data suggest the existence of distinct loci affecting BP and arterial stiffness in Dahl salt-sensitive hypertensive rats. Moreover, as found with increasing number of traits [7, 21–25], arterial stiffness portrays sex-specificity implying the existence of differential genetic mechanisms underlying vessel stiffening in male and female Dahl salt-sensitive rats. The subsequent identification of genes underlying the aortic pulse wave velocity and strain QTLs will define the genetic basis of aortic stiffness in the Dahl salt-sensitive hypertensive rat model. This will provide a critical first step towards the elucidation of novel aortic-stiffness genetic mechanisms, and provides the basis for further study of aortic stiffness genes as putative genetic biomarkers of adult-onset hypertension and its target-organ complications.

## Methods

This study was performed in strict accordance with the recommendations in the Guide for the Care and Use of Laboratory Animals of the National Institutes of Health. The protocol was approved by the Committee on the Ethics of Animal Experiments of Boston University School of Medicine (Permit Number: AN-14966). All measurements were performed under isoflurane anesthesia, and every effort was made to minimize suffering. Euthanasia was done by exsanguination and removal of vital tissue under anesthesia. Secondary euthanasia procedure of pneumothorax was also done under anesthesia (isoflurane or ketamine/xylazine at 40–80 mg/kg ketamine + 5–20 mg/kg xylazine). This method is consistent with the recommendation of the Panel of Euthanasia of the American Veterinary Medical Association (AVMA http://grants.nih.gov/grants/guide/notice-files/NOT-OD-01-029.html).

### Genetic crosses

Inbred Dahl S/jrHsd and Dahl R/jrHsd rats were obtained from Harlan (Indianapolis, Indiana). Parental strains (Dahl R/jrHsd x Dahl S/jrHsd) were crossed to produce F1 progeny. The F2 subjects were derived from brother-to-sister mating of F1 hybrids to produce the F2 female (*n* = 140) and F2 male (*n* = 142) segregating populations. Animals were maintained on Purina 5001 (0.4 % NaCl) rat diet from inception.

### Arterial stiffness measurements

We performed high-resolution ultrasonography measurement of pulse wave velocity (PWV) in rat abdominal aorta (average distance = 1.0 cm) essentially as described [6]. PWV was defined as Δd/Δt, distance (d) between two points divided by the difference in transit time (t) of the pressure wave arrival at said two points. The transit time was defined as the time from the peak of the ECG R-wave to the foot of the velocity upstroke [26], with the foot defined as the point at the end of diastole when the steep rise of the wave front begins [27]. Measurement of strain (D^SYS^ – D^DIAS^)/D^DIAS^, where D = diameter of artery, was also done in abdominal aorta as another measure of arterial stiffness and distensibility. Each measurement of PWV and strain represents the average of five independent determinations per subject. We characterized 12 Dahl R, 10 Dahl S, 142 F2 males and 140 F2 females at six weeks of age. We also measured PWV in 10 Dahl R and 9 Dahl S rats at three weeks of age. Animals were not phenotyped for blood pressure. The ultrasound micro-imaging and Doppler ultrasound studies were done with 0.8 % isoflurane anesthesia – hence expected to not induce non-physiological artifacts since it is less than the 1 % isoflurane anesthesia level defined as “baseline level” for coronary blood flow with heart rates similar to sleeping mice [28] and documented to not alter aortic impedance [29].

### Intercross linkage analysis

Phenotype distributions were analyzed for normality; data transformations were done when necessary and datasets that passed Kolmogorov-Smirnov normality testing (SigmaPlot 11.2) were used for linkage analysis. Specifically, the current QTL analysis was performed using Log 10 [aortic PWV] and untransformed aortic Strain as quantitative traits. Data analysis was not controlled for BP since at six weeks of age Dahl S and Dahl R rats have equivalent BP [30], therefore not likely to be an independent confounder of the arterial stiffness measurements. Linkage maps, marker regression and composite interval mapping were done with the Map Manager QTXb19 (MMQTXb19) program for windows [31] which generates a likelihood ratio statistic (LRS) as a measure of the significance of a possible QTL. Genetic distances were calculated using Kosambi mapping function (genetic distances are expressed in centiMorgan, cM). Critical significance values (LRS values) for interval mapping were determined by a permutation test (2000 permutations at all loci tested) on our male and female cohorts using Kosambi mapping function and a dominant, recessive or additive regression model. Values for significant linkage LRS = 13.8 (LOD 3.0) and for highly significant linkage LRS = 19.8 (LOD 4.3). LRS 4.6 delineates LOD 1-support interval. Confidence interval for a QTL location was estimated by bootstrap resampling method wherein histogram single peak delineates the QTL and peak widths define confidence interval for the QTL. We also performed interaction analysis using the Map Manager QTXb19 program applying a two-stage test paradigm for determination of interaction in which the pair of loci must pass two tests in order to be reported as having a significant interaction effect. First, the significance of the total effect of the two loci must be < 0.00001 and second, the pairs of loci must exhibit a *P* value < 0.01 for the interaction effect.

### Genotyping

DNA purification from spleen tissue samples was done by using the DNeasy Blood & Tissue Kit (QIAGEN) following manufacturer’s instructions. SNP genotyping was carried out on an Applied Biosystems 7900 Real-Time PCR System. SNPs (*n* = 98) and SSLP markers (*n* = 12) were identified as informative markers between our parental strains by using SNPlotyper from the Rat Genome Database. SNP assays (TaqMan assays) were procured from Applied Biosystems and were validated in our laboratory.

### Statistical analyses

We performed one-way ANOVA followed by all pairwise multiple comparisons using the Holm-Sidak Test for PWV and Strain measurements as indicated per experimental comparison.

## Abbreviations

Dahl S: Dahl salt-sensitive
Dahl R: Dahl salt-resistant
QTL: quantitative trait locus
LOD: logarithm of the odds score
PWV: pulse wave velocity
CYP11B2: cytochrome P450, family 11, subfamily B, polypeptide 2
NOS3: nitric oxide synthase 3 (endothelial cell)
AS: arterial stiffness
BP: blood pressure
ACVR1: Activin A receptor, Type 1
VEGF-A: Vascular endothelial growth factor A
ADAMTS1: A Disintegrin And Metalloproteinase with thrombospondin type 1 motif, 1
SNP: single nucleotide polymorphism
SSLP: single strand length polymorphism
Chr: chromosome
M: male
F: female
pmF: post-menopausal female
Mbp: mega base pairs
cfPWV: carotid-femoral pulse wave velocity
FURIN: paired basic amino acid cleaving enzyme
IGF1R: insulin-like growth factor-1 receptor
MEF2A: myocyte-specific enhancer factor 2A
CHSY1: chondroitin synthase 1
PACE4: proprotein convertase subtilisin/kexin type 6
ADD2: β-adducin
TACR1: neurokinin-1 receptor
ADRA2B: α-2B adrenergic receptor
IL6: interleukin-6
3′-BCL11B: 3′-B-cell CLL/lymphoma 11B (zinc finger protein)

## References

[1] Ge D, Young TW, Wang X, Kapuku GK, Treiber FA, Snieder H (2007). Heritability of arterial stiffness in black and white American youth and young adults. Am J Hypertens.

[2] Mitchell GF, DeStefano AL, Larson MG, Benjamin EJ, Chen MH, Vasan RS (2005). Heritability and a genome-wide linkage scan for arterial stiffness, wave reflection, and mean arterial pressure. The Framingham Heart Study. Circulation.

[3] Lacolley P, Challande P, Osborne-Pellegrin M, Regnault V (2009). Genetics and pathophysiology of arterial stiffness. Cardiovasc Res.

[4] Gaugier D, Behmoaras J, Argoud K, Wilder SP, Pradines C, Bihoreau MT (2005). Chromosomal mapping of QTL controlling elastin content in rat aorta. Hypertension.

[5] Behmoaras J, Osborne-Pellegrin M, Gauguier D, Jacob MP (2005). Characteristics of the aortic elastic network and related phenotypes in seven inbred rat strains. Am J Physiol Heart Circ Physiol.

[6] Herrera VL, Decano JL, Giordano N, Moran AM, Ruiz-Opazo N (2014). Aortic and carotid arterial stiffness and epigenetic regulator gene expression changes precede blood pressure rise in stroke-prone Dahl salt-sensitive hypertensive rats. PLoS One.

[7] Herrera VL, Tsikoudakis A, Ponce LR, Matsubara Y, Ruiz-Opazo N (2006). Sex-specific QTLs and interacting-loci underlie salt-sensitive hypertension and target-organ complications in Dahl S/jr^HS^ hypertensive rats. Physiol Genomics.

[8] Herrera VL, Pasion KA, Moran AM, Ruiz-Opazo N (2012). Differential genetic basis for pre-menopausal and post-menopausal salt-sensitive hypertension. PLoS One.

[9] Border WA, Noble NA (1994). Transforming growth factor beta in tissue fibrosis. New Engl J Med.

[10] Robertson AK, Rudling M, Zhou X, Gorelik L, Flavell RA, Hansson GK (2003). Disruption of TGF-beta signaling in T cells accelerates atherosclerosis. J Clin Invest.

[11] Mallat Z, Tedgui A (2002). The role of transforming growth factor beta in atherosclerosis: novel insights and future perspectives. Curr Opin Lipidol.

[12] Roberts AB, Sporn MB, Assoian RK, Smith JM, Roche NS, Wakefield LM (1986). Transforming growth factor type beta: rapid induction of fibrosis and angiogenesis in vivo and stimulation of collagen formation in vitro. Proc Natl Acad Sci U S A.

[13] Grainger DJ, Metcalfe JC (1995). A pivotal role for TGF-beta in atherogenesis?. Biol Rev Camb Philos Soc.

[14] Topper JN (2000). TGF-beta in the cardiovascular system: molecular mechanisms of a context-specific growth factor. Trends Cardiovasc Med.

[15] Jacob T, Hingorani A, Ascher E (2005). Overexpression of transforming growth factor-beta1 correlates with increased synthesis of nitric oxide synthase in varicose veins. J Vasc Surg.

[16] Li M, Scott DE, Shandas R, Stenmark KR, Tan W (2009). High pulsatility flow induces adhesion molecule and cytokine mRNA expression in distal pulmonary artery endothelial cells. Ann Biomed Eng.

[17] Jonsson-Rylander AC, Nilsson T, Fritsche-Danielson R, Hammarstrom A, Behrendt M, Andersson JO (2005). Role of ADAMTS-1 in atherosclerosis: remodeling of carotid artery, immunohistochemistry, and proteolysis of versican. Arterioscler Thromb Vasc Biol.

[18] Dolan JM, Sim FJ, Meng H, Kolega J (2012). Endothelial cells express a unique transcriptional profile under very high wall shear stress known to induce expansive arterial remodeling. Am J Physiol Cell Physiol.

[19] Dolan JM, Meng H, Sim FJ, Kolega J (2013). Differential gene expression by endothelial cells under positive and negative streamwise gradients of high wall shear stress. Am J Physiol Cell Physiol.

[20] Luque A, Carpizo DR, Iruela-Arispe ML (2003). ADAMTS1/METH1 inhibits endothelial cell proliferation by direct binding and sequestration of VEGF_165_. J Biol Chem.

[21] Glorioso N, Herrera VL, Bagamasbad P, Filigheddu F, Troffa C, Argiolas G (2007). Association of *ATP1A1* and *Dear* SNP-haplotypes with essential hypertension: sex-specific and haplotype-specific effects. Circ Res.

[22] Herrera VL, Decano JL, Bagamasbad P, Kufahl T, Steffen M, Ruiz-Opazo N (2008). Sex-specific hippocampus-dependent cognitive deficits and increased neuronal autophagy in DEspR haploinsufficiency in mice. Physiol Genomics.

[23] Herrera VL, Bagamasbad P, Decano JL, Ruiz-Opazo N (2011). AVR/NAVR deficiency lowers blood pressure and differentially affects urinary concentrating ability, cognition and anxiety-like behavior in male and female mice. Physiol Genomics.

[24] Herrera VL, Pasion KA, Tan GA, Moran AM, Ruiz-Opazo N (2013). Sex-specific effects on spatial learning and memory, and sex-independent effects on blood pressure of a < 3.3 Mbp rat chromosome 2 QTL region in Dahl salt-sensitive rats. PLoS One.

[25] Glorioso N, Herrera VL, Didishvili T, Ortu MF, Zaninello R, Fresu G (2013). Sex-specific effects of NLRP6/AVR and ADM loci on susceptibility to essential hypertension in a Sardinian population. PLoS One.

[26] Williams R, Needles A, Cherin E, Zhou YQ, Henkelman RM, Adamson SL (2007). Noninvasive ultrasonic measurement of regional and local pulse-wave velocity in mice. Ultrasound Med Biol.

[27] Zhang X, Greenleaf JF (2006). Noninvasive generation and measurement of propagating waves in arterial walls. J Acoust Soc Am.

[28] Hartley CJ, Reddy AK, Madala S, Michael LH, Entman ML, Taffet GE (2008). Doppler estimation of reduced coronary flow reserve in mice with pressure overload cardiac hypertrophy. Ultrasound Med Biol.

[29] Reddy AK, Namiranian K, Looyd EE, Bryan RM, Taffet GE, Hartley CJ (2009). Effect of isoflurane on aortic impedance in mice. Conf Proc IEEE Eng Med Biol Soc.

[30] Kong JQ, Taylor DA, Fleming WW (1995). Sustained hypertension in Dahl rats. Negative correlation of agonist response to blood pressure. Hypertension.

[31] Manly KF, Cudmore RH, Meer JM (2001). Map Manager QTX, cross-platform software for genetic mapping. Mamm Genome.

[32] Mitchell GF, Verwoert GC, Tarasov KV, Isaacs A, Smith AV, Yasmin (2012). Common genetic variation in the 3′-BCL11B gene desert is associated with carotid-femoral pulse wave velocity and excess cardiovascular disease risk. The AortaGen Consortium. Circ Cardiovasc Genet.

